# A novel cocktail therapy based on quintuplet combination of oncolytic herpes simplex virus-2 vectors armed with interleukin-12, interleukin-15, GM-CSF, PD1v, and IL-7 × CCL19 results in enhanced antitumor efficacy

**DOI:** 10.1186/s12985-022-01795-1

**Published:** 2022-04-22

**Authors:** Han Hu, Siqi Zhang, Linkang Cai, Haixiao Duan, Yuying Li, Junhan Yang, Yang Wang, Biao Liu, Shuang Dong, Zhizheng Fang, Binlei Liu

**Affiliations:** 1 National ‘‘111’’ Center for Cellular Regulation and Molecular Pharmaceutics, Key Laboratory of Fermentation Engineering (Ministry of Education), Hubei Provincial Cooperative Innovation Center of Industrial Fermentation, College of Bioengineering, Hubei University of Technology, Wuhan, China; 2 Wuhan Binhui Biopharmaceutical Co., Ltd., Wuhan, China; 3 Hubei Cancer Hospital, Tongji Medical College, Huazhong University of Science and Technology, Wuhan, China

**Keywords:** Oncolytic herpes simplex virus, Combined therapy, 4T1, CT26

## Abstract

**Background:**

Selectively replicating herpes simplex virus-2 (HSV-2) vector is a promising treatment for cancer therapy. The insertion of multiple transgenes into the viral genome has been performed to improve its oncolytic activity.

**Methods:**

Herein, we simultaneously constructed five “armed” oncolytic viruses (OVs), designated oHSV2-IL12, -IL15, GM-CSF, -PD1v, and IL7 × CCL19. These OVs delete the *ICP34.5* and *ICP47* genes with the insertion of transgenes into the deleted *ICP34.5* locus. The anti-tumor efficacy in vivo was tested in the syngeneic 4T1 and CT26 tumor-bearing mice model.

**Results:**

The OVs showed comparable oncolytic capability in vitro. The combination therapy of oHSV2-IL12, -IL15, GM-CSF, -PD1v, and IL7 × CCL19 exhibited the highest tumor inhibition efficacy compared with the treatment of single OV or two OVs combination.

**Conclusions:**

The OVs armed with different transgenes combination therapy also named 5-valent oHSV2 (also called cocktail therapy) might be an effective therapeutic strategy for solid tumors.

**Supplementary Information:**

The online version contains supplementary material available at 10.1186/s12985-022-01795-1.

## Background

Oncolytic viruses (OVs) are considered as a novel type of immunotherapy for cancer patients. OVs conditionally replicate in cancer cells, causing cell lysis. The malignant cell death induced by OVs is perceived as a form of immunogenic cell death (ICD), due to the release of danger-associated and pathogen-associated molecular patterns (DAMPs and PAMPs), and tumor-associated antigens (TAA) as well. The process offers various immune signals to activate the anti-tumor innate and adaptive immune system [1, 2]. Accordingly, OVs have been developed as vectors to express different immunomodulatory transgenes to improve the potential oncolytic efficacy [3–5].

Oncolytic herpes simplex virus (oHSV) are promising OVs which have been under investigation in clinical trials against numerous tumors, including melanoma, glioma, breast, and pancreatic cancers [6, 7]. Talimogene laherparepvec (T-VEC), the first oHSV1, was approved by the U. S. FDA for advanced melanoma therapy in 2015 [8]. In the previous study, we constructed oHSV2, with deletion of gene *ICP34.5* and *ICP47* and insertion of *GM-CSF*, and investigated its efficacy in various tumor-bearing mouse models [9]. Preclinical studies showed that oHSV2 was stable at the genetic and biological levels [10]. The therapeutic effect of oHSV2 was also investigated on mouse colon carcinoma. The results showed that oHSV2 treatment reshaped the tumor immune microenvironment, with the reduction of inhibitory immune cells [regulatory T cells (Tregs) and myeloid-derived suppressor cells (MDSCs)] and increment of positive immune cells [CD8^+^ T, natural killer (NK), and dendritic cells (DCs)] [9]. A multicenter phase I/II clinical trial aimed to assess the safety, biodistribution, and oncolytic efficacy of oHSV2 in advanced solid tumor patients was initiated [11]. Results exhibited that intratumoral OH2 injection was well tolerated, and showed long-lasting antitumor activity in metastatic rectal and esophageal cancer patients [11].

The ability to carry large and/or various transgenes in the viral genome is one of the advantages of HSV vectors [12]. In addition to direct lyse the tumor cells by replicating within the tumors, HSV can elicit a systemic antitumor immune response as well [13]. Thus, immunomodulatory molecule genes are often inserted into oncolytic HSV vectors to improve its anti-tumor activity. We previously developed oncolytic oHSV2 vectors, which encode different immunomodulators: anti-PD-1 antibody and IL-15. Anti-PD-1 antibody expressing oHSV2-aPD1 enhances the antitumoral efficacy of oHSV2 in the B16R melanoma mouse model which is poorly immunogenic [14]. Compared with the parent vector of oHSV2, IL-15 expressing oHSV2-IL15 also showed better antitumoral efficacy both in vitro and in the xenograft mouse model [15].

In this study, the five OVs integrated with *IL-15*, *IL-12*, *PD-1v*, *IL-7* × *CCL19*, or *GM-CSF* were used alone or in combination to compare the oncolytic effect. In the CT26 colon and 4T1 breast tumor model, the cocktail therapy of the combined five armed OVs (1/5 dose each) exhibited the greatest anti-tumor efficacy and led to complete eradication of implanted tumors.

## Methods

### Cell lines and mice

Cell lines used in this research contained CT26 (mouse colon carcinoma cell), B16F10 (mouse melanoma cell), B16R (mouse melanoma cell), A549 (human lung carcinoma cell), 4T1 (mouse mammary carcinoma cell), BGC823 (human gastric cancer cell). B16R was constructed from B16F10 integrated with the HSV receptor and kept in our lab [16]. Vero, B16R, B16F10, BGC823 and 4T1 cells were cultured with a DME/F-12 medium containing 10 % fetal bovine serum (FBS). A549 and CT26 were incubated with medium RPMI-1640 containing 10 % FBS. The cells mentioned above were incubated in the 37°C, 5 % CO2 incubator.

Six-week-old female Balb/c mice were purchased from Hubei Center for Disease Control and Prevention (Hubei, China). The animals were raised in a specific pathogen-free (SPF) environment. All animal experiments were approved by the Animal Ethics Committee of Hubei University of Technology (HBUT No. 2019011). Animals were offered ad libitum access to water and food in a controlled environment with a 12 h light/dark cycle.

### Virus construction

oHSV2-PD1v was constructed based on oHSV2, which is engineered from the HG52 strain as reported before [17]. Transfer plasmid pHG52d34.5-DC-PD1v and pHG52d34.5-CMV-eGFP were used to engineer oHSV2-PD1v by two rounds of homologous recombination. pHG52d34.5 contains the downstream and upstream flanking region of gene *ICP34.5*. The CMV-eGFP sequence, cloned from pcDNA3.1-CMV-eGFP was inserted into the area between the upstream and downstream regions of pHG52d34.5 to get pHG52d34.5-CMV-eGFP. Thereafter, the shuttle plasmid was cotransfected with the HG52 genome into Vero cells to construct oHSV2-eGFP by homologous recombination. Then, oHSV2-eGFP was purified through six rounds of plaque assay under the fluorescent microscope. Then, shuttle plasmid pHG52d34.5-DC-PD1v was used to substitute the eGFP cassette with the same process, leading to recombinant OV oHSV2-PD1v. The *PD1v* gene sequence was disclosed in Table 1. All the shuttle plasmids pHG52d34.5-CMV-eGFP and pHG52d34.5-DC-PD1v were acquired with standard cloning technique and confirmed by sequencing. Other recombinant OVs oHSV2-IL12 and IL7 × CCL19 were constructed with the same strategy mentioned above. IL7 × CCL19 meant IL7 and CCL19 were linked with foot and mouth disease virus 2A peptide sequence for oHSV2- IL7 × CCL19 to express the two genes concomitantly [18]. The final recombinant OVs were amplified with Vero cells, titrated, split into equal aliquots. Then, the viruses were stocked under − 80°C before usage.

**Table 1:** Estimated sequence and protein expression of the target gene

Virus	Target gene (bp)	Protein expressed (pg/mL)
oHSV2-GM-CSF	1000	21,771.92 ± 934.50
oHSV2-IL12	2420	2281.78 ± 25.17
oHSV2-PD1v	1471	–
oHSV2-IL15	1910	224^[16]^
oHSV2-IL7 × CCL19	1511	1370.63 ± 20.38 (IL7); 2063.30 ± 98.13 (CCL19)

### DNA Ladder, enzyme-linked immunosorbent assay and western blot assay

OVs with MOI = 0.1 was used to infect Vero cells. 48 h post-infection, the cells were collected and lysed in DNAzol (BioTeke Corporation, China). Thereafter, DNA was gathered and washed with ethanol (75 %). Then amplifications were performed and the PCR products were observed with agarose gel electrophoresis (1 %).

The quantity of IL-12 expressed by oHSV2-IL12 was identified by enzyme-linked immunosorbent assay (ELISA) in Vero cells. Briefly, Vero cells cultured in flask T75 were infected with oHSV2-IL12. After 48 h, the amount of IL-12 in the supernatant was analyzed using IL-12 ELISA kit (R&D Systems, YD419-05) according to the instruction. IL-7 ELISA kit (R&D Systems, M7000) and CCL19 ELISA kit (Abcam, ab242236) were used to detect the related cytokines expressed by oHSV2- IL7 × CCL19 with the similar method described above. GM-CSF concentration was detected according to the sandwich ELISA method which has been described before [19].

All the samples were extracted from oHSV2-PD1v or mock-infected Vero cells with Pierce™ IP Lysis Buffer contained protein inhibitor. The protein sample was resolved by SDS-PAGE and transferred onto a polyvinylidene fluoride (PVDF) membrane. The antibody (Ab) with an HRP-conjugated anti-PD1v IgG Fc was purchased from Abcam (Abcam, ab97225).

### Virus infection and killing assay in vitro

For oncolytic spectrum investigation, human and mouse tumor cells, including B16R, A549, and CT26 were cultured overnight in a six-well plate with the concentration of 4 × 10^5^ cells per well. Thereafter, the cells were infected with OVs with MOI = 1. Subsequently, the OVs mediated tumor cells killing process was observed for 48 h by the PerkinElmer Operetta imaging system. To test the killing ability of OVs mediated through PBMC in vitro, CT26-GFP cells (1 × 10^4^ cells/well) were seeded overnight in 96-well plates. Then OVs were inoculated with MOI = 0.1. Subsequently, the PBMC were isolated by from the mice and added onto the CT26-GFP tumor cells. The killing of tumor cells by PBMC was monitored for 60 h with the PerkinElmer Operetta imaging system. Cancer cell lysis kinetics was analyzed by an xCELLigence real-time cell analyzer (ACEA Biosciences, U. S.) as reported. 1 × 10^4^ cancer cells were inoculated into the E-plate. 24 h post culture, OVs (MOI = 1) or media were supplemented. Cell index was noted every 20 min.

### Tumor models

For the CT26 Balb/c mouse model, CT26 (1 × 10^6^ cells per mouse) in 100 μL were subcutaneously injected into the right flank of Balb/c mice. When the mean tumor diameter came to 100 mm^3^, the mice were intratumorally inoculated with PBS (100 μL) or OVs (100 μL). All the OVs (HSV2-IL12, -PD1v, -IL15, -IL7 × CCL19, -GM-CSF) were diluted into 1 × 10^7^ CCID_50_/mL in advance. In the *5voHSV2* (cocktail therapy) or the two combination treatment group, the OVs were mixed together in equal volume. CCID_50_ assay was also performed to confirm that every treatment containing the OVs with the same quantity. Tumor diameter was measured by the caliper. The formula used to determine tumor volume (mm^3^) was as follows: [(major axis) × (minor axis)^2^ × 0.5]. For the rechallenge experiment, mice “cured” by oHSV (complete response) were inoculated with CT26 cells (1 × 10^6^ cells per mouse) in the contralateral flank. Treatment-naïve mice were also included in the experiment to confirm the tumor growth. And the tumor growth was measured by caliper.

For the 4T1 Balb/c mouse model, 4T1 (1 × 10^6^ cells/mouse) in 100 μL were subcutaneously inoculated into the Balb/c mice with the right flank. When the average tumor diameter came to 100 mm^3^, the mice were intratumorally inoculated with PBS (100 μL) or OVs (100 μL). Tumor diameter was observed by an electric caliper. The formula used to determine tumor volume (mm^3^) was as follows: [(major axis) × (minor axis)^2^ × 0.5]. For histological evaluation, parenchymal organs (heart, liver, spleen, lung, kidney, cerebrum, and cerebellum) were dissected from 10 mice (5 mice in the control group and 5 mice treated with *5voHSV2*). Then the samples were formalin fixed and paraffin embedded (FFPE). 4 mm tissue sections were prepared and stained with hematoxylin and eosin. Images were obtained with Olympus (Tokyo, Japan).

### Statistics

Unless otherwise stated, quantitative data are exhibited as mean ± SEM. Statistical data were analyzed by Student t-test. Mice survival was shown using Kaplan–Meier survival curve. Differences were considered significant at *P* < 0.05 (*P < 0.05; **P < 0.01; ***P < 0.001; ****P < 0.0001); ns, not significant.

## Result

### Comparison of anti-tumor activity between oncolytic herpes simplex virus type I and type II

**Fig. 1:**
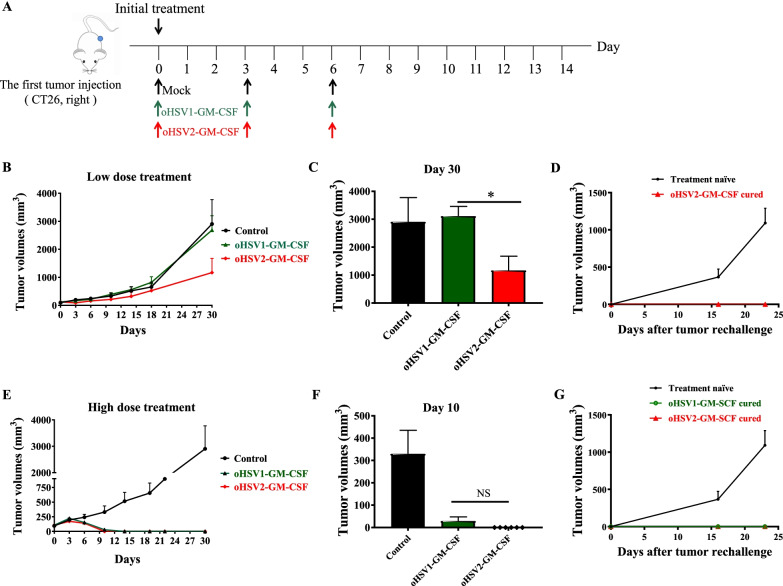
Therapeutic effects of Oncolytic simplex virus type I and II in a CT26 model. Balb/c mice were subcutaneously implanted with CT26 tumor cells and intratumorally injected with oHSV1-GM-CSF and oHSV2-GM-CSF (n = 5–6 per group). **A** Schematic representation of the CT26 tumor-bearing mice model treated with the OVs on days 0, 3, 6. **B** Tumor volumes growth curves of the low dose (1 × 10^4^ CCID50) and mock-treated groups, and data are presented as the mean + SD. **C** The tumor volumes on day 30 of the mice from the control and OVs treated groups in (**B**). *P < 0.05 by unpaired Student’s *t*-test. **D** Tumor volumes growth curves of treatment-naive and mice cured with the low dose treatment rechallenged by subcutaneous injection of CT26 cells. *n* = 2 for oHSV2-GM-CSF cured group, *n* = 4 for treatment-naive group. **E** Tumor volumes growth curves of the high dose (1 × 10^6^ CCID50) and mock-treated groups, data are shown as mean + SD. **F** The tumor volumes on day 10 of the mice from the control and OVs treated groups in (**D**). **G** Tumor volumes growth curves of treatment-naive and mice cured with the high dose treatment rechallenged by subcutaneous injection of CT26 cells. *n* = 6 for oHSV2-GM-CSF cured group, *n* = 6 for oHSV1-GM-CSF cured group, and *n* = 4 for treatment-naive group

It was reported that HSV-2 oncolytic virus FusOn-H2 showed better activity in killing MDA-MB-435 human breast tumor cells under a lower multiplicity of infection (MOI), leading to more tumor-free mice [20]. The results indicated that HSV-2 may have better oncolysis activity than HSV-1. In order to further evaluate the antitumoral efficacy of oHSV1 and oHSV2, we established a CT26 Balb/c mouse colon cancer model. The tumor-bearing mice were intratumorally injected with PBS, oHSV2-GM-CSF or oHSV1-GM-CSF since tumor average volume came to 100 mm^3^ (Fig. 1A). A low dose (1 × 10^4^ CCID50) and a high dose (1 × 10^6^ CCID50) of OVs were used in this experiment. In the period of treatment and observation, no tumor regression was found in the control group. In the low dose treatment groups, on day 10 after the initial inoculation, one mouse in the oHSV2-GM-CSF therapy group showed tumor regression. Afterward, the number of tumor regression mice in this group constantly expanded. Two mice in the oHSV2-GM-CSF inoculation group were completely tumor-free. In the oHSV1 treatment or control groups, no tumor regression could be found. On day 30 since the initial inoculation, the mean tumor volume in the control group was 2 905 mm^3^, while the mean tumor volumes of the mice inoculated with oHSV2-GM-CSF and oHSV1-GM-CSF were 1 168 mm^3^ and 3 112 mm^3^, respectively. The mean tumor volume difference between the oHSV2-GM-CSF and oHSV1-GM-CSF inoculation group was significant (*P* < 0.05) (Fig. 1C).

High-dose virus inoculation of oHSV1-GM-CSF and oHSV2-GM-CSF both inhibited tumor growth (Fig. 1E, F). On day 10 after the first treatment, all the mice in the oHSV2-GM-CSF therapy group showed tumor regression. On day 10 after the first treatment, the mean tumor volume in the group of control was 330 mm^3^, while the mean tumor volumes in the group inoculated by oHSV2-GM-CSF or oHSV1-GM-CSF were 0 mm^3^ and 29 mm^3^, respectively (Fig. 1F).

In order to study the effect of oncolytic virus therapy on tumor-specific immune memory, we challenged 14 tumor-free mice (low dose treatment group of oHSV2, n = 2; high dose treatment group of oHSV1, n = 6; high dose treatment group of oHSV2, n = 6) from Fig. 1B, E by subcutaneous injection of CT26 cells into the opposite side. In treatment-naïve mice, tumors grew with predictable kinetics. But in mice previously cured by oHSV1-GM-CSF or oHSV2-GM-CSF, tumors failed to grow (Fig. 1D, G). The results indicated that both oHSV1-GM-CSF and oHSV2-GM-CSF treatment could elicit potent endogenous antitumor immunity.

### Construction and characterization of the oncolytic herpes simplex virus 2 vectors that encode different cytokines.

Based on the results above, oHSV2 was used as the backbone to construct different recombinant oncolytic viruses for further research. oHSV2 was constructed with the deletion of *ICP34.5* and *ICP47* genes. Neurovirulence gene *ICP34.5* deletion could enhance the tumor selectivity of oHSV2. *ICP47* deletion could increase the expression of MHC class I in infected cells, promoting the presentation of TAAs. Meanwhile, *ICP47* deletion enhances the expression of gene *US11*, which improves oncolytic activity.

**Fig. 2:**
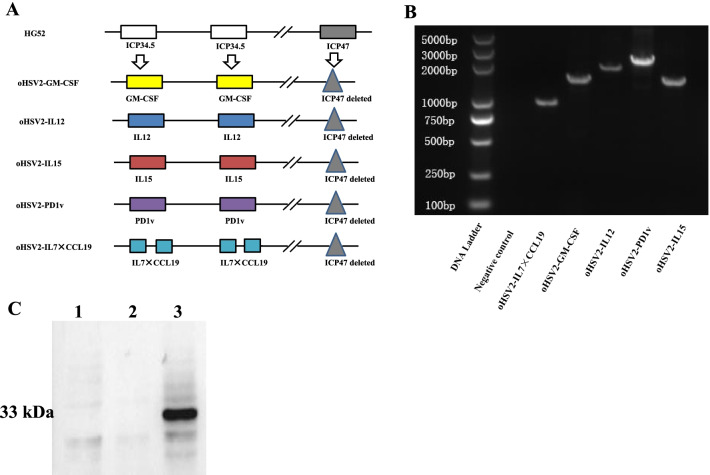
Schematic of the construction and characterization of oHSV2-IL12, -PD1v, -IL15, -IL7 × CCL19, GM-CSF. **A** The oHSV2 vectors were constructed based on the HG52 strain. Modifications include ICP47 and ICP34.5 genes deletion and the insertion of the related transgenes. IL7 × CCL19 means that IL7 and CCL19 were linked through the 2A peptide. **B** PCR identification of the inserted sequences. Lane M: marker, lane 1: negative control, lane 2–6 are oHSV2-IL7 × CCL19, GM-CSF, IL12, PD1v and IL15, respectively. **C** Western blot identification of PD1v (approximately 33 kDa) expression in oHSV2-PD1v infected Vero cells. Lane 1, mock-treated control, lane 2, oHSV2-GFP treated control, lane 3, oHSV2-PD1v infected Vero cells

Recombinant oncolytic viruses HSV2-IL12, -PD1v, -IL15, -IL7 × CCL19, and -GM-CSF that expressed cytokines IL12, PD1v, IL15, IL7, CCL19, and GM-CSF were constructed from oHSV2 (Fig. 2A). The coding sequences for these chemokines were listed in Table 1. And these genes were all introduced into the ICP34.5 locus (Fig. 2A). The inserted sequences were amplified by PCR from the recombinant virus respectively (Fig. 2B). Compared with the estimated target sequence listed in Table 1, it was confirmed that the target sequence has been inserted into the viral genome (Fig. 2B). Vero cells were infected with oHSV2-IL12, -PD1v or -IL7 × CCL19 and supernatants were collected for ELISA assay. The results indicated that infected cells expressed the relative cytokines (Table 1). As there was not commercial ELISA kit for PD1v detection, western blot was performed to confirm the PD1v expression of oHSV2-PD1v (Fig. 2C).

Thus, these results proved that recombinant virus oHSV2-IL12, -PD1v, and -IL7 × CCL19 have been successfully constructed and infection with the recombinant virus led to the detectable cytokines expression.

### Determination oncolytic spectrum and cell lysis induced by different recombinant oncolytic viruses in vitro

**Fig. 3:**
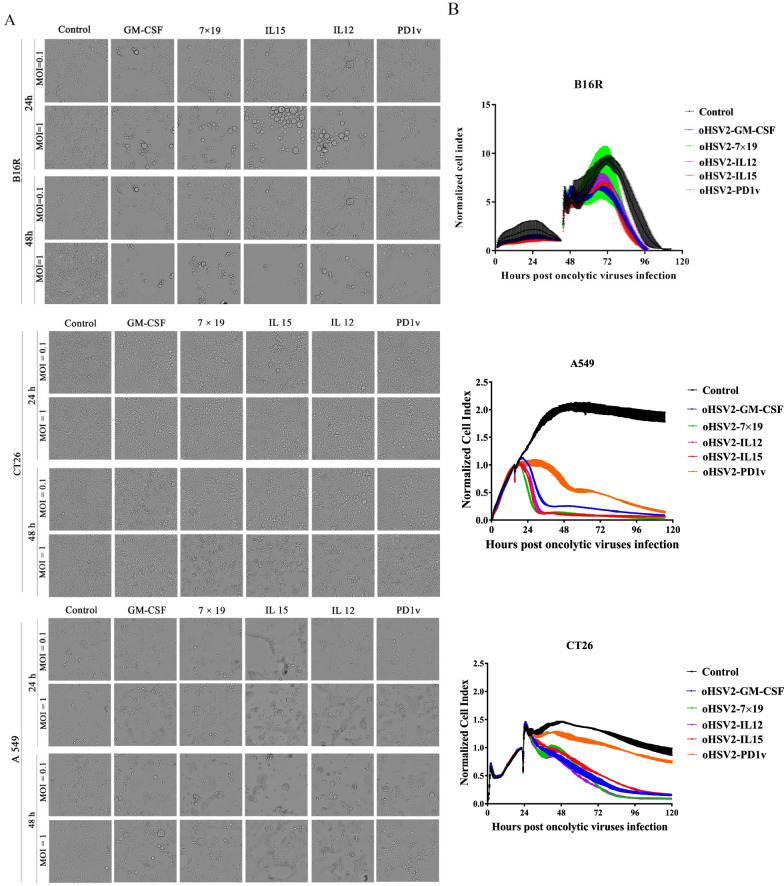
Infectious and oncolytic activities of oHSV2-IL12, -PD1v, -IL15, -IL7 × CCL19, GM-CSF in vitro. **A** the recombinant viruses were used to infect tumor cell lines, containing B16R, A549, and CT26 at MOI = 1 for 48 h. Photographs were collected at 24 h and 48 h post-infection. **B** real-time xCELLigence cell analyzer was used to monitor the kinetics of tumor cell lysis induced by the recombinant viruses. Means of cell index from duplicate wells are presented. Data are representative of three experiments

The oncolytic and infectious activities of different cytokines expression oncolytic viruses were performed to evaluate the effect of different transgenes on virus characteristics. Microscopy observation also showed that B16R, A549, and CT26 cells were both well infected with the different recombinant viruses (Fig. 3A), suggesting that the constructed viruses had a similar infection spectrum in the cell lines tested.

We evaluated the influence of insertion of cytokine transgenes on the lytic activity of the recombinant viruses with real-time cell analysis (Fig. 3B). Tumor cell lines B16R, A549, and CT26 were tested. Results showed that under MOI = 1, these recombinant viruses demonstrated similar oncolytic abilities in B16R and A549. However, when CT26 was used, oHSV2-PD1v induced slower lysis compared with other recombinant viruses (Fig. 3B).

### Inhibition of tumor growth in vivo by mono-therapy and *5voHSV2*

**Fig. 4:**
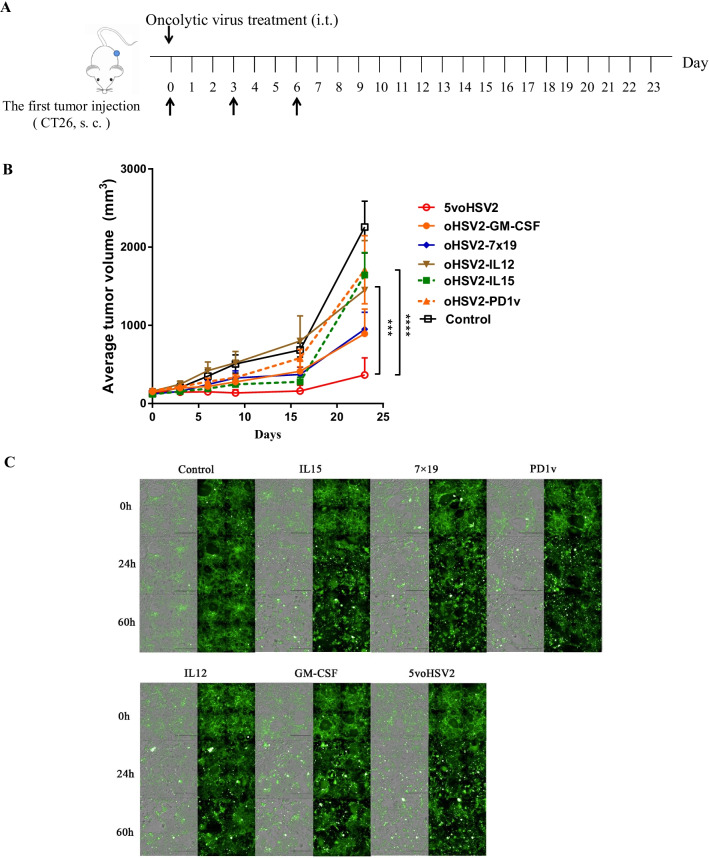
Therapeutic effects of mono virus and *5voHSV2* treatment in a CT26 Model. Balb/c mice were subcutaneously implanted with CT26 tumor cells and intratumorally inoculated with OVs on days 0, 3, 6 (n = 8–10 per group). **A** Schematic depiction of the experiment. **B** The tumor volume growth curves of the OVs and mock-treated groups, the data are shown as mean + SD. ***P < 0.001, ****P < 0.0001 (vs *5voHSV2* group) by repeated-measures 2-way ANOVA with Bonferroni’s correction. **C** Mouse PBMC killing of CT26-GFP tumor cells. CT26-GFP cells were seeded in the 96-well plate overnight. Then infected with blank control, oHSV2-IL12, -PD1v, -IL15, -IL7 × CCL19, GM-CSF, and *5voHSV2* (MOI = 0.1). PBMC were added with effector cells:target cells ratio being 2:1 for another 60 h. The killing activity was recorded with the PerkinElmer Operetta imaging system

The data described above arouse examination of the antitumor activity of the different recombinant OVs therapy in vivo. Subcutaneous CT26 tumor-bearing BalB/c mice model was used to assess the therapeutic efficacy. The tumor-bearing mice were treated with PBS (mock), oHSV2 encoding IL15, IL12, PD1v, IL7, CCL19, GM-CSF, or a combination of the five different viruses (*5voHSV2*) on days indicated in Fig. 4A by intratumoral inoculation when the tumor mean volumes reached 100 mm^3^ (Fig. 4A). Treatment with the OVs delayed the tumor progression (Fig. 4B). On day 24 after treatment, mice treated with *5voHSV2* had an average tumor volume of 362 mm^3^, while the average tumor volume of mice inoculated with OVs was 1 450 mm^3^ (oHSV2-IL12), 1 711 mm^3^ (oHSV2-PD1v), 951 mm^3^ (oHSV2-IL7 × CCL19), 893 mm^3^ (oHSV2-GM-CSF) and 1 443 mm^3^ (oHSV2-IL15). Altogether, our data showed that the *5voHSV2* treatment showed better inhibition activity than any other virus treatment alone (Fig. 4B). To evaluate the cytokines biological activity in terms of PBMC stimulation in vitro, CT26-GFP cells were cocultured with mouse PBMC for 60 h after the OVs inoculation (MOI = 0.1) (Fig. 4C). *5voHSV2* treatment showed better tumor cells killing activity mediated by PBMC when compared with other groups.

### Inhibition of tumor growth in vivo by *5voHSV2*

**Fig. 5:**
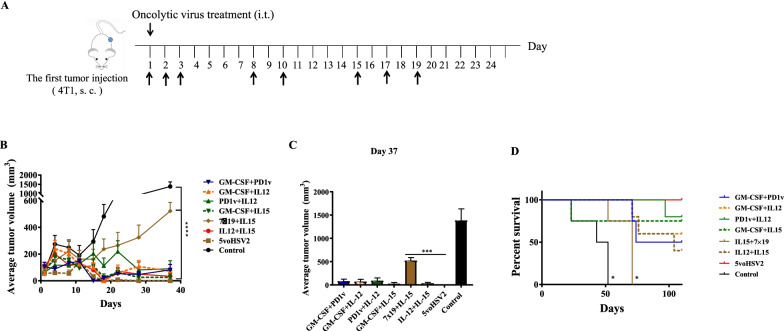
Therapeutic effects of cocktail therapy in the 4T1 model. Balb/c mice were subcutaneously implanted by 4T1 tumor cells and intratumorally injected with OVs on days 1, 2, 3, 8, 10, 15, 17, 19 (n = 4–5 per group). **A** Schematic depiction of the experiment. **B** Tumor volume growth curves of the OVs and mock-treated groups, data are shown as mean + SD. ***P < 0.001 (vs *5voHSV2* group) by repeated-measures 2-way ANOVA with Bonferroni’s correction. **C** The mice tumor volumes on day 37 from the control and OVs treated groups in (**B**). ***P < 0.001 by unpaired *t*-test. **D** Survival is shown by Kaplan–Meier analysis. *P < 0.05 (vs. *5voHSV2* group) by log-rank test

To determine whether these cytokines combined together would improve antitumoral efficacy, we assessed tumor growth and mice survival rates in the 4T1 mouse model. The tumor-bearing mice were intratumorally injected by PBS (mock) or different OVs combinations on days indicated in Fig. 5 when the tumor mean volumes came to 100 mm^3^ (Fig. 5A). The *5voHSV2* treatment showed the strongest inhibitory effect on tumor growth (Fig. 5). In this group, all animals had a complete response, and the tumor was no longer palpable since day 18. For survival analyses, mice were monitored for 110 days since 4T1 cells were inoculated in the right flank. All the treatments with different virus combinations improved survival time. And the *5voHSV2* therapy exhibited the best antitumor activity (Fig. 5C). No obvious damage could be observed in the main organ tissues from the mice treated with the *5voHSV2* (Additional file 1: Fig. S1).

**Fig. 6:**
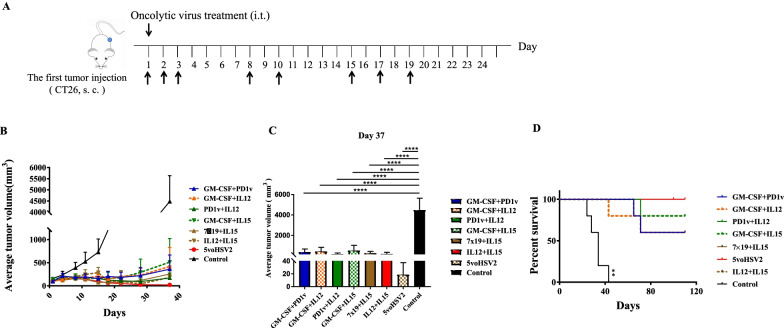
Therapeutic effects of cocktail therapy in the CT26 model. Balb/c mice were subcutaneously implanted with CT26 tumor cells and intratumorally injected with OVs on days 1, 2, 3, 8, 10, 15, 17, 19 (n = 5 per group). **A** Schematic depiction of the experiment. **B** Tumor volume growth curves of the OVs and mock-treated groups, data are shown as mean + SD. **C** The tumor volumes of mice from the control and OVs treated groups on day 37 from (**B**). ****P < 0.0001 by 1-way ANOA with Tukey’s post hoc test. **D** Survival is monitored with Kaplan–Meier analysis. **P < 0.01 (vs. *5voHSV2* group) by log-rank test

To further confirm the inhibition effect of cocktail therapy, we also monitored tumor growth and survival rates in the CT26 mouse model treated with the *5voHSV2*. The tumor-bearing mice were intratumorally injected with PBS or the combination of different viruses on days as indicated when the tumor mean volumes arrived 100 mm^3^ (Fig. 6). The greatest tumor inhibition activity was also seen in the *5voHSV2* therapy group (Fig. 6). For survival analyses, mice were tracked for 110 days since the day that CT26 inoculated in the right flank. All the treatments with different virus combinations increased survival time. And *5voHSV2* showed the strongest therapeutic effect (Fig. 6C). The data above indicated that cocktail therapy showed better inhibition activity than any other different combination therapy or monotherapy. Cocktail therapy might be a potential regime for tumor therapy.

## Discussion

Oncolytic viral therapy is an emerging candidate treatment for cancer. The OVs could selectively infect and directly result in carcinoma cell death. Moreover, the killing process promotes innate and adaptive immune responses to apoptotic tumor cells. As a result, OVs could lead to lasting immune response, eliminating the existing tumors, preventing metastatic and relapsed tumors [21]. Since 2015, T-VEC was gradually approved for advanced melanoma therapy in U. S., Europe, and Australia [22, 23]. The study and application of OV immunotherapy have made substantial progress, announcing more probabilities and challenges as well. Herpes simplex virus vectors have been developed into a multi-functional oncolytic platform which allows for efficient expression of cytokines, prodrug convertases, and target genes. According to our acknowledgements, combination therapy with different recombinant herpes simplex viruses has not been reported previously. Our translational pilot study provides preliminary evidence supporting that the novel cocktail therapy based on quintuplet combination of oHSV-2 vectors integrated with five different cytokines might be an effective strategy for cancer treatment.

The antitumor immune response induced by oHSV plays an important role in antitumor activities in vivo, which support the insertion of immunostimulatory genes. Here, we constructed different immunostimulatory genes expression OVs, and these OVs infected eukaryotic cells expressing related cytokines. Through virus infection experiment and real-time cytolytic assay, it was indicated that the integration of the genes into the oHSV2 backbone would not change the oncolytic spectrum, nor hinder the cytolytic activity. In vitro data demonstrate the successful construction, transgene expression and lysis of human and mouse cancer cells of these recombinant viruses. Interestingly, the differences in cell killing kinetics between cell lines may reflect the differences in the biology of the single clonal tumor cells.

Then, we evaluated the efficacy of HSV-2 vectors expressing IL-12, IL-15, PD-1v, GM-CSF, and IL7 × CCL19 using syngeneic CT26 tumor-bearing model. The result showed that these OVs had a similar anti-tumor activity with oHSV2-GM-CSF and oHSV2-IL7 × CCL19 being a litter better than the other three viruses. Previous research has showed that T-zone fibroblastic reticular cells secreted IL-7 and CCL19 are required for the T-cell zone formation and maintenance in lymphoid organs. A recent study also revealed that the incorporation of 7 × 19 into CAR-T cells significantly enhanced the antitumor activity against human solid tumor [24]. These findings indicated that oHSV2-IL7 × CCL19 might inhibit the tumor growth through T cell recruit and activation. It was reported that NV1042, an oncolytic HSV vector armed with IL-12, showed better efficacy than a GM-CSF armed vector of the same backbone (NV1034) in certain tumor-bearing mice models [25]. Shanawaz et al. also reported that an oncolytic HSV carrying cytokine interleukin 12 (surnamed G47Δ-mIL12) could significantly reduce primary tumor growth and metastasis in the 4T1 syngeneic TNBC model [26]. However, in our CT26 tumor-bearing mice model oHSV2 armed with GM-CSF showed better efficacy than IL-12 armed vector of the same backbone. This might be caused by the different tumor-bearing models or different replication capacity of the viruses.

Nowadays, various reports have indicated that the antitumor actions of OVs were dependent on tumor cell lysis/necrosis induced by OV multiplication and immune response induced by the lysed cells [27]. The antitumor immune response triggered by OVs plays a more important role than the direct oncolytic effect in maintaining a lasting antitumor effect [28]. One of the most important advantages of using oHSV2 platform is that it enables us to investigate the combination therapy effect of the various armed OVs. According to our knowledge, the combination therapy of five oHSV2 vectors carrying different immunostimulatory genes has not been reported before. Some groups have tried the combination therapy of different cytokines expressing OVs. Mamoru et al. reported that IL-7 and IL-12 expression of recombinant vaccinia viruses increased tumor-infiltrating lymphocytes in the LLC lung cancer model, leading to a better tumor therapy efficacy than IL-12 alone. And they demonstrated that IL-7 and IL-12 combination virotherapy could increase the intratumoral CD8^+^ T cells clonality and anti-tumor response rate as well [29]. The synergistic effect of IL-12 and IL-18 has also been investigated. Intratumoral expression of one combined with systemic injection of the other would improve the antitumor effect with better safety, while the systemical inoculation of IL-12 and IL-18 would cause severe toxicity with a steep increase of IFN-γ in serum [30–32]. Another advantage about cocktail therapy is that intratumoral delivery of the cytokines with oHSV2 would restrict expression to the tumor compartment and might reduce the side effects that would occur under systemic inoculation.

The data showed that the quintuplet combination of oHSV2-IL12, -IL15, GM-CSF, PD1v, and IL7 × CCL19 demonstrated the best efficacy among the single oHSV2 vectors or two vectors combination against CT26 or 4T1 in vivo. The *5voHSV2* showed stronger antitumor activities than two OVs combination in vivo and improved the mouse survival rate, leading to “cure” the animals. Consistent with the previous research, the rechallenge models exhibited that cocktail therapy results in a systemic lasting antitumor immune memory while directly lysing tumor cells. The tumor-specific immune response finally prevented secondary identical tumorigenesis. The results indicated that the improved efficacy might result from the interaction of the five immunostimulatory genes expressed that may have worked favorably to activate antitumor responses of the adaptive immune system. However, unlike replication-defective viral or non-viral vectors, it is difficult to elaborate the specific anti-tumor mechanism of the cocktail therapy, especially different cytokines expressing oHSV2 might have different characteristics of replication, spreading, and host immune stimulation.

## Conclusions

In summary, our results suggest that the combined therapy of multiple oncolytic HSV-2 vectors carrying different immunostimulatory genes would be a potential strategy for tumor therapy. We believe that the development of oHSV-2 vector is of great significance not only to increase the therapeutic effect but also to cope with the broad variety of progression stages, tumor types, etc. The cocktail therapy strategy merits further investigation clinically.

## Supplementary Information

**Additional file 1: Fig. S1**. Histopathological analysis of murine organ tissues by hematoxylin and eosin (H&E) staining.

## Data Availability

All relevant data are included in the manuscript, supplementary material or can be obtained from the authors on reasonable request.

## Abbreviations

OVs: Oncolytic viruses
HSV2: Herpes simplex virus 2
TAA: Tumor-associated Antigen
