# Supplementary material for: A novel cocktail therapy based on quintuplet combination of oncolytic herpes simplex virus-2 vectors armed with interleukin-12, interleukin-15, GM-CSF, PD1v, and IL-7 × CCL19 results in enhanced antitumor efficacy
Source: Virol J. 2022 Apr 22;19:74. doi: 10.1186/s12985-022-01795-1 (PMC9034647; doi:10.1186/s12985-022-01795-1)
Supplement: Supplementary file 1 — Additional file 1: Fig. S1. Histopathological analysis of murine organ tissues by hematoxylin and eosin (H&E) staining. [file 12985_2022_1795_MOESM1_ESM.pdf]

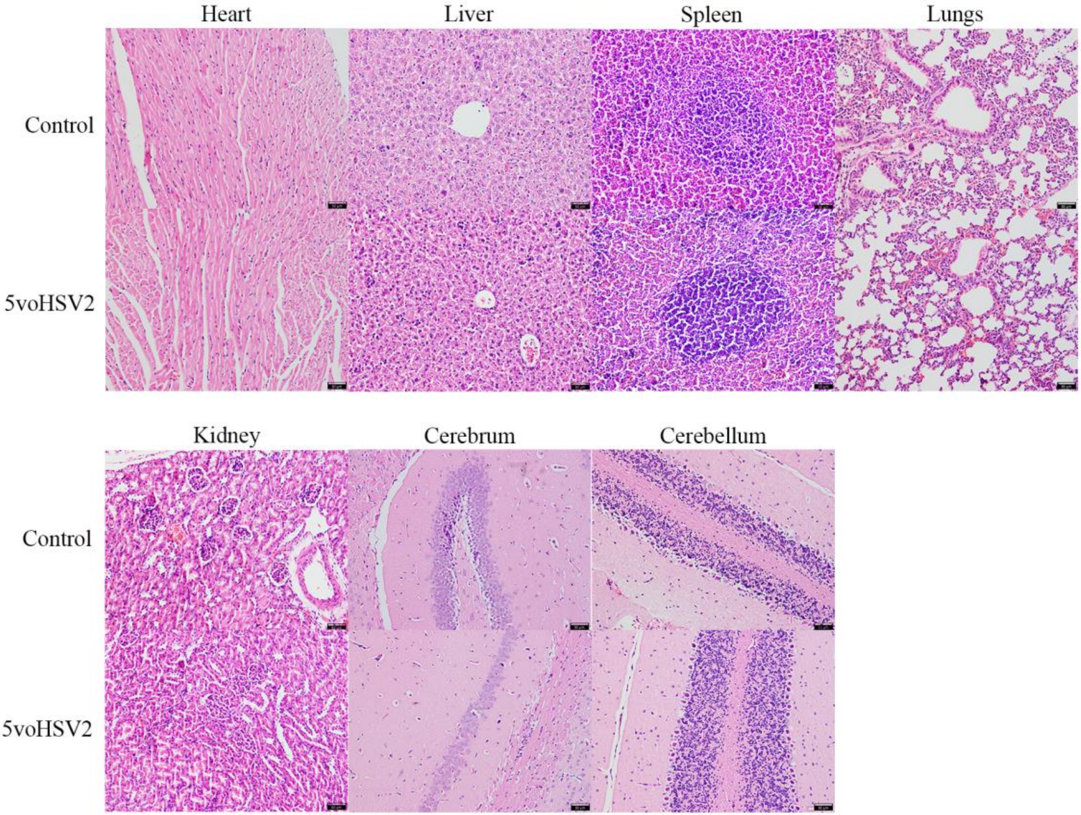

Figure S1. Histopathological analysis of murine organ tissues by hematoxylin and eosin (H&E) staining. Mice were mock treated (n = 5) or with *5voHSV2* (n=5) and sacrificed on day 7 following treatment. Different tissues were harvested, formalin-fixed, paraffinembedded, and stained with H&E. Representative photomicrographs are shown. The images were taken with a BX53 microscope and a camera under magnification ×20. Each scale bar represents 50 μm.
